# A simulation-based framework for tracking decision-making development in youth basketball: operationalising Vygotsky’s zone of proximal development through Bayesian multilevel modelling and verbal protocol analysis

**DOI:** 10.3389/fspor.2026.1872966

**Published:** 2026-08-06

**Authors:** Humberto M. Carvalho, Caio G. Miguel, Carlos E. Gonçalves

**Affiliations:** 1 Department of Health Sciences, Faculty of Medicine, Lund University, Lund, Sweden; 2 School of Sports, Federal University of Santa Catarina, Florianópolis, Santa Catarina, Brazil; 3 Post-graduate Programme in Psychology, Federal University of Santa Catarina, Florianópolis, Santa Catarina, Brazil; 4 Faculty of Sport Sciences and Physical Education, University of Coimbra, Coimbra, Portugal

**Keywords:** activity theory, basketball, coaching pedagogy, tactical learning, verbal protocols, Vygotsky, youth development

## Abstract

**Introduction:**

Sports coaching research remains polarised between direct instruction approaches emphasising feedback and repetition vs. constraints-led approaches favouring athlete discovery through task design. This debate persists partly because existing methods cannot distinguish whether athletes are genuinely internalising tactical understanding or merely following external instructions. We present a methodological simulation study proposing a framework that operationalises Cultural-Historical Activity Theory—particularly Vygotsky’s Zone of Proximal Development (ZPD)—through Bayesian multilevel modelling and verbal protocol analysis to track the transition from coach-dependent to self-directed tactical thinking in youth athletes. No human participants were involved, as this study uses exclusively simulated data.

**Methods:**

We outline a research protocol integrating: (1) a four-phase longitudinal design (baseline → scaffolded coaching → independent test → transfer challenge), (2) verbal protocol instruments assessing spatial awareness, decision-making reasoning, and metacognitive reflection, (3) linguistic coding distinguishing other-regulation from self-regulation, and (4) Bayesian multilevel models capturing individual developmental heterogeneity. Using simulated data (n=80 athletes, 25 sessions), we demonstrate—not empirically test—that the framework’s statistical model recovers theoretically-predicted developmental patterns embedded in the data-generating process. We additionally simulate a “compliance” data-generating process, in which scaffolding gains do not persist after withdrawal, to test whether the framework can discriminate internalisation from mere compliance.

**Results:**

The simulation confirms that, under specified assumptions, the framework successfully recovers embedded theoretical patterns: accelerated growth during scaffolding, maintained gains after withdrawal, successful transfer, differential ZPD width effects, and progression from external to internal regulation. We report this recovery result from a single simulated draw, and separately report whether the same modelling pipeline can discriminate this internalisation pattern from a simulated “compliance” alternative in which scaffolding gains do not persist. A full simulation-based calibration exercise across many replications would strengthen the recovery claim further but is disproportionate to a methodological proof-of-concept and is left for future methodological work.

**Discussion:**

We provide researchers with a complete methodological package—study design templates, scoring rubrics, coding schemes, statistical specifications, and reproducible code—enabling rigorous empirical tests of Activity Theory in sport. This proof-of-concept bridges Vygotskian theory with computational methods and establishes that the proposed framework has adequate sensitivity to detect theoretically-predicted patterns when they are present in real data.

## 1 Introduction

The specialization process in basketball is a long and complex one. It starts around 12 years of age and goes through adolescence and early adulthood, with the athlete facing successive hurdles in terms of training volume and competitive levels. It is during this period of time that the player, whilst maturing biologically and socially, learns the skills required to perform at increasingly higher levels. Those skills integrate the fundamentals of motor execution with the capacity to “see and read” the game, mandatory to respond to dynamic and unpredictable match situations [1].

Hence, the readiness to play on- and off-the-ball combines technical abilities with adequate decision-making. Existing literature based on coaching experience provides abundant information about contents and sequencing for players’ development across the targeted age span. However, if the technical training planning is relatively straightforward, there are still gaps and zones of obscurity regarding how to improve awareness about spacing and timing on- and off-the-ball but also how to teach and refine decision-making in the presence of teammates and opponents.

It is well known that high-level performance demands not only exceptional athletic capacity and technical proficiency, but also the tactical knowledge necessary to make the right choices under match pressure [2]. But if repetition in practice drills provides designed neuro-muscular stimuli that improve motor responses, it is hard to evaluate the way players internalise and modify their understanding of the game in order to modify their behaviour when facing real competitive challenges.

This apparent gap in our knowledge on how adolescent players in specialising settings learn and internalise tactical instructions and their own game experiences arises from practical and theoretical hurdles. Indeed, coaches can observe, assess and manipulate their athletes’ path to attain higher stages of expertise over extended periods of training, but that process is time- and resources-consuming and implies methodological steps and arrangements that few coaches can carry alone.

### 1.1 Theoretical landscape

From a theoretical point of view, researchers and coaches (via education programmes) have been dealing with a simplistic dichotomy between the so-called traditional methods and the constructivist (Teaching GAmes for Understanding, Game Sense, Athlete-Centred Approach, etc.) or more functionalist trends, like Constraints-Led Approaches and Ecological Dynamics [3–5]. The former postulates that instruction, feedback and extensive repetition are crucial to a robust and sustainable technical and tactical learning. The latter claims that the athletes must be active learners, with their progress being stimulated and monitored through encouraging and questioning by the coach.

We argue that we face a pedagogical problem. If the “traditional” training methods make coaches comfortable about their job, they seem to fail to grasp the social, cultural and educational world views of contemporary young athletes. By contrary, “active” styles seem to answer poorly to the needs of athletes who try to perform in competitive environments and attain elite levels.

We also argue that social activity is a decisive contributor to cognitive, emotional and motor development, particularly for the tactical and decision-making skills that are the focus of this study; we do not claim that social mediation is the sole determinant of development, since growt and maturity- related factors are also well documented, especially in the physical fitness performance [6, 7]. Furthermore, there are no theorems in Basketball to explain the perfect pass or the perfect cut to the basket, resulting that Basketball skills are complex cultural activities. Therefore, it is imperative to go further in researching the ways players learn, which means to go beyond descriptive models and build, intentionally, models that are able to make complex phenomena understandable for participants and capable of anticipating the consequences of the activity through expanded learning [8].

### 1.2 Debates in coaching pedagogy

Coaching research has been characterised by paradigmatic divisions—researchers defending theoretical camps rather than testing mechanisms. The field exhibits three overlapping debates that our study directly addresses.

#### 1.2.1 Instruction vs. discovery

Traditional coaching emphasises direct instruction: coaches demonstrate, athletes replicate, feedback corrects errors. This approach assumes efficient skill transfer through explicit teaching and deliberate practice [2]. Extensive drill repetition with corrective feedback is considered the cornerstone of skill development. Critics argue this produces “robots who can execute drills but can’t read games” [4]—athletes who follow instructions but lack autonomous decision-making capacity under competitive pressure.

Constraints-Led Approaches and Ecological Dynamics counter that athletes learn best by discovering solutions through manipulated practice constraints [5]. Coaches design representative tasks that preserve the information-action coupling of competition and ask questions rather than provide answers [9]. Proponents claim that direct instruction creates coach-dependent athletes who cannot transfer learning to novel situations and that implicit learning through exploration produces more robust, adaptable skill. We note that this tradition is not *unguided* in any literal sense: the constraints-led and ecological-dynamics literatures [e.g., Newell’s model of constraints; [5]] rest on the deliberate design of task, environmental and individual constraints, which constitutes a form of indirect guidance rather than its absence. Where we use the shorthand “minimal guidance” to describe this pole, we mean minimal direct/explicit instruction, not the absence of pedagogical design.

We argue this is a false dichotomy that misunderstands the social nature of learning. Vygotsky theorised that cognitive development requires social mediation—neither pure instruction nor pure discovery optimizes learning [1], a theoretical claim that the present framework is designed to make empirically testable, not one we treat here as already established. The question is not *whether* to coach but *how* coaching transforms from external regulation to internal agency. Both extremes fail: continuous direct instruction without withdrawal maintains dependency, whilst discovery learning pursued without any scaffolding ignores the Zone of Proximal Development (ZPD) where scaffolding accelerates learning.

#### 1.2.2 Performance outcomes vs. cognitive process

A methodological divide separates researchers tracking performance outcomes (game statistics, win percentages, skill execution tests) from those examining learning processes [10]. The researchers tracking performance outcomes focus on objective evidence: “Does it improve performance?” Performance metrics provide clear, quantifiable benchmarks and resist subjective interpretation. The researchers examining learning processes argues performance metrics are downstream consequences that don’t reveal *how* athletes learn or *why* some coaching works [11]. Understanding mechanisms enables principled intervention rather than trial-and-error coaching.

These views often talk past each other. Performance researchers dismiss verbal protocols and think-aloud methods as subjective, post-hoc rationalizations. Process researchers argue that focusing solely on outcomes leads to atheoretical “what works” pragmatism without understanding transferability or individual differences.

Our framework integrates both perspectives. Verbal protocols capture cognitive sophistication (process) whilst growth modelling tracks developmental trajectories (outcome). We can test whether tactical understanding correlates with performance improvement and whether certain cognitive profiles predict learning rates. The integration allows us to ask: Does internalisation (observable through linguistic self-regulation) predict performance gains and transfer to novel challenges?

#### 1.2.3 Evidence-based practice vs. individualized coaching

Sports coaching science seeks generalizable principles from population-level research [12]. Randomized controlled trials, meta-analyses, and large-sample studies aim to identify “what works” on average so coaches can implement evidence-based practices. However, coaches observe marked individual differences: identical training produces different results [13]. Some athletes thrive under high-volume training whilst others plateau or burn out. Some internalise tactical concepts rapidly whilst others require extended scaffolding.

This has led to calls for *precision coaching*—individualized interventions based on athlete profiles [14]. Critics counter that chasing individual differences prevents accumulation of reliable coaching knowledge. If every athlete is unique, what generalizable principles can inform coaching education?

Fixed- and random-effects (multilevel) models are an established statistical approach [15], not a new resolution of this epistemological debate—the present contribution is in what they let us report jointly, not in the modelling technique itself. Fixed effects estimate population-level patterns (e.g., “On average, scaffolding accelerates learning by factor X”). Random effects quantify individual deviations (e.g., “an athlete may show 3× faster growth than average during scaffolding; note this is a shrunk, prior-dependent estimate, illustrative of what the model reports rather than a free-standing finding”). Coaches receive both generalizable insights *and* athlete-specific information from the same model [16]. We use “synthesis” here in this specific, narrower sense: both quantities are estimated jointly from one model. This does not resolve the underlying tension between generalising across athletes and describing any one athlete individually—doing so would require real individual differences to follow the model’s assumed Gaussian form, which we cannot verify from simulated data. This is not a compromise—it is a synthesis recognising that both population patterns and individual heterogeneity are real and measurable.

#### 1.2.4 Our contribution to these debates

Rather than defending one paradigm, we operationalise Activity Theory to empirically test mechanisms underlying the debates:

- Does scaffolded coaching accelerate learning more than independent practice? (Debate 1)
- Do verbal protocols provide evidence beyond performance data? (Debate 2)
- Can we model both population patterns and individual heterogeneity? (Debate 3)

By using simulated data with known parameters, we demonstrate the framework can detect these patterns when ground truth is known, including—critically—that it can distinguish an internalisation pattern from a mere-compliance pattern when both are simulated. This provides confidence in the methodological tools itself: that the models are correctly specified and sensitive to the distinctions the theory cares about. It is not, and cannot be, confidence that Activity Theory’s predictions are true of real athletes, since that question is precisely what remains to be tested empirically. Researchers can then apply it empirically and test which coaching practices actually produce internalised tactical understanding rather than mere compliance or performance improvements that don’t transfer.

### 1.3 Cultural-historical activity theory: a framework for understanding learning in Sport

Vygotsky founded cultural-historical psychology and, with it, the ZPD construct used throughout this paper [1, 17]. Activity Theory proper was subsequently established by Leontiev [18], who introduced activity as its central category, and extended by Engeström and Sannino [8] into the activity-system and expansive-learning frameworks. Clot’s [19] work on activity clinics represents a related development within the broader post-Vygotskian tradition, drawing more directly on Leontiev and on dialogical approaches to language and work. We follow this lineage rather than attributing Activity Theory’s authorship to Vygotsky himself, while retaining the ZPD as our organising construct.

Two concepts from this broader tradition are central to our framework:

1. The concept of *Zone of Proximal Development*: The ZPD is the distance between what a learner can do independently and what they can achieve with guided support [1]. This concept helps us understand when and how coaching intervention accelerates learning—not all athletes benefit equally from instruction at all times.
2. The unit-of-analysis principle, in Vygotsky’s original methodological sense, requires identifying the smallest analytic unit that still retains the properties of the whole under study (his own example being word meaning as the unit of verbal thought) [17]. We adopt this principle operationally: in our design, the verbal protocol response—rather than an isolated action or an isolated score—is the unit we treat as retaining both the individual’s tactical reasoning and its embeddedness in the coach-mediated activity. Where the wider activity-system literature examines the relations between individual and collective action explicitly [8], our present design is scoped to the individual athlete’s verbal protocols; we do not analyse team-level activity structures here, and we note this as a boundary of the current framework rather than describing it as our unit of analysis.

Importantly, Activity Theory predicts that cognitive development proceeds through *internalisation*—the transformation of socially mediated activity (coach instructions, peer advice, environmental cues) into internal psychological processes [20]. In coached sport contexts, this manifests as progression from external, coach-directed problem-solving to internal, self-directed decision-making. A basketball player initially relies on coach prompts—“Where is the defender? What space is open?”—but gradually internalizes this questioning framework, spontaneously asking themselves these questions without external cues.

### 1.4 The training artifact

The choice of the training artifact (exercise, drill) does not result from questioning, but from the coach’s pedagogical priorities. This initial unbalance can be corrected by the appropriation of the artifact by the players, or by the introduction of cognitive artifacts by the coach. The repetition of the training situation will lead to a spiral of development that includes the two sides of the activity: (i) the external performance, which is observable by the coach and the peers; (ii) the internal transformation, that can only be inferred.

The proposed experimental situation is *1 on 1 with a passer*, where the offensive player receives a pass under defensive pressure and tries to beat the defender through intelligent decision-making. The drill is simple enough to allow for the targeted parameters to be categorised and measured and sufficient representative of a competitive game event. The experiment is repeated in 25 training sessions; the coach can intervene to help the players and peer interaction is also allowed.

Our knowledge about the system allows us to set priors with heuristic and instrumental characteristics that pave the way to the manipulation of the system parameters. Besides the simulation parameters—Age, Tactical Awareness, Technical Skills and Learning Capacity, that are coded, measured at baseline and at the end of the experiment—we use a Verbal Protocol which represents the unit of analysis at the level of the individual athlete, namely how the players make sense, over time, of their perceptions about spatial awareness and decision-making strategies.

We should be explicit about the epistemic status of this approach. The first and third authors have extensive applied coaching and talent-development experience in basketball, and this domain expertise directly informed our judgement of what parameter values (growth rates, effect sizes, heterogeneity) are ecologically plausible. This “insider” position is best understood as a double-edged resource rather than a pure asset. On one hand, it grounds otherwise arbitrary simulation choices in field experience. On the other hand, it creates a specific risk of confirmation bias and motivated reasoning: because we set the parameters the model is later shown to recover, a demonstration built entirely on our own priors cannot, by itself, adjudicate whether those priors are correct—it can only show that the statistical machinery faithfully returns what was put into it. Making the code, priors and generative assumptions fully available (see Data and Code Availability) allows this position to be inspected and challenged; it does not, on its own, resolve the risk.

### 1.5 Hypotheses from activity theory

In line with Activity Theory [18, 19] and addressing the coaching pedagogy debates outlined above, we hypothesize that:

1. temporary scaffolding within the ZPD accelerates the internalisation of tactical decision-making—moving athletes from rule-following to autonomous agency—beyond what independent practice alone can achieve;
2. tactical awareness mediates the capacity to perform;
3. the players’ search for help, from coaches or from peers, depends of their perceived capacity to perform (testing ZPD predictions);
4. improvements in declarative knowledge or verbal sophistication are positively correlated with improvements in performance capacity (testing whether process measures predict outcomes).

These hypotheses allow us to test competing claims: if instruction-only approaches are correct, continuous scaffolding should produce maximal learning; if discovery-only approaches are correct, Phase 1 independent practice should show learning rates comparable to Phase 2 scaffolding. Vygotskian theory predicts neither extreme—instead, temporary scaffolding within ZPD (Phase 2) should accelerate learning beyond independent practice (Phase 1), but gains should be maintained after withdrawal (Phase 3) only if internalisation occurred, not if athletes were merely complying with external instructions.

We acknowledge that it is very difficult to discern if the artifact acted as a mere exercise or if it became a significant activity that makes sense for the player, allowing him/her to go from a rule-follower to an agent of tactical choices. The illumination of the transition from a training drill to a significant activity with sense represents the conceptual core of our scientific enquiry.

### 1.6 Analytical challenges

The option for an experimental design, with a clear definition of variables, or parameters, the need of coding and plausible description of latent classes that can be measured raises important analytical challenges. The collaboration and agreement between researchers and coaches about specific terminology and evaluation criteria is essential.

Traditional approaches to studying cognitive development in sport face several limitations:—How do we capture internal cognitive transformation, not just external performance?—How do we model athletes who start at different levels and learn at different rates?—How do we formally test whether scaffolding accelerates learning, whether gains are maintained after support is withdrawn, and whether learning transfers to new challenges?—How do we account for multiple sources of social mediation (coach, peers, environment)?

#### 1.6.1 The present study: a methodological framework

This study demonstrates how Bayesian multilevel modelling can operationalise Activity Theory principles to track cognitive development in youth basketball. Our purpose is to formalise a research approach and explore whether it is possible to map or trace developmental outcomes systematically. We use simulated data to validate that the framework works—the results themselves are not substantive findings but demonstrations of methodological capabilities. We state explicitly that this is a methodological demonstration and not a ready-to-deploy athlete assessment tool: no verbal protocol instrument or linguistic coding scheme reported here has been validated on real athletes, and none should be used to make individual coaching decisions before that validation is carried out.

## 2 Materials and methods

### 2.1 Study design: four developmental phases

We simulate 80 players (ages 12–16) across 25 training sessions organised into four phases aligned with Vygotsky’s Zone of Proximal Development (Table 1). This is achieved through systematic manipulation of scaffolding and task difficulty across 25 training sessions. The four-phase structure allows us to test specific predictions from Activity Theory: that scaffolding accelerates learning (Phase 2); that learning is internalised and maintained after scaffolding withdrawal (Phase 3); and that internalised understanding transfers to more challenging situations (Phase 4). Specifically, baseline session set independent practice, with no coaching, to establishe starting points. The scaffolded learning sessions with progressive coaching support (directive → questioning → minimal cues) are set to create optimal learning conditions. The internalisation test sessions are independent practice at original difficulty to test whether learning persists without support. The transfer challenge sessions consist of independent practice at higher difficulty to test whether understanding applies to new situations. After each session, verbal protocols assess three dimensions of tactical cognition: (i) *Spatial awareness*—“What do you look for when receiving the ball?”; *Decision-making reasoning*—“How do you decide which skill to use?”; *Metacognitive reflection*—“What have you learned about creating advantages?”. Each phase serves a distinct theoretical purpose in tracking cognitive development from external coach-directed thinking to internal self-directed reasoning. Table 1 summarizes the design, including the Vygotskian predictions we aim to test in each phase.

**Table 1:** Four-phase study design operationalizing the zone of proximal development.

Phase	Sessions	Scaffolding	Task difficulty	Vygotskian prediction
Phase 1: Baseline	1–5	None (independent practice)	Standard	Minimal growth—task is challenging without support
Phase 2: Scaffolded learning	6–15	Sessions 6–8: Directive (explicit instructions)	Standard	Strongest cognitive growth—athletes operate within ZPD with appropriate support
		Sessions 9–12: Questioning (Socratic prompts)		
		Sessions 13–15: Minimal cues (brief reminders)		
		Intensity: 1.0→0.3		
Phase 3: internalisation test	16–20	None (complete withdrawal)	Standard	Maintained growth but slower rate—learning has been internalised from external to internal regulation
Phase 4: transfer challenge	21–25	None (independent)	Increased (faster defender, reduced space)	Renewed growth—internalised framework applies to novel challenges at higher difficulty

*Phase 1 (Baseline)* establishes each athlete’s starting cognitive sophistication and natural learning rate without coaching intervention. Athletes perform the exercise independently with only initial task instructions, allowing us to measure what they can accomplish on their own—the lower boundary of Vygotsky’s Zone of Proximal Development.

*Phase 2 (Scaffolded Learning)* creates optimal conditions for cognitive development by providing systematic coaching support that gradually decreases in intensity. This phase is the longest (10 sessions) to allow sufficient time for learning within the ZPD. The gradual withdrawal of scaffolding follows Vygotsky’s principle that support should be provided only when needed and removed as learners become more capable.

During Phase 2, coaching support was systematically manipulated across three levels:

- Sessions 6–8 (Directive scaffolding): Coaches provided explicit instructions such as “Check the defender’s hip position—if they’re square you can go either way”
- Sessions 9–12 (Questioning scaffolding): Coaches shifted to Socratic prompts: “What do you see about the defender’s positioning? Where is the space opening up?”
- Sessions 13–15 (Minimal cues): Coaches offered only brief reminders: “Read and decide”

We quantified scaffolding intensity on a continuous scale from 1.0 (maximum directive support at Session 6) declining linearly to 0.3 (minimal cues at Session 15). We report this quantification as part of the design rationale, but we do not use it as a separate covariate in the statistical model reported below (Table 2). Because intensity follows a fixed, deterministic schedule that is identical for every athlete and declines linearly with time-in-Phase-2, it has no variance independent of session number within Phase 2: intensity and time-in-phase are the same variable read in opposite directions, and a model cannot separate “the effect of scaffolding intensity” from “the effect of practising longer within Phase 2.” A genuine dose-response test would require randomising intensity across athletes or sessions, which the present design does not do. Phase 2’s fixed effect ($\beta_{2}$, Table 2) should therefore be read as the combined effect of practising within Phase 2 under a declining-intensity schedule, not as isolating intensity’s independent contribution.

**Table 2:** Bayesian ordinal multilevel model specification.

Component	Conceptual description	Statistical notation	Interpretation
Outcome variable	Verbal protocol score (ordinal: 0–12)	$Y_{ij}∼\mathrm{OrderedProbit}(\mu_{ij},\tau )$	Probability of each score category for athlete i at session j
Fixed effects: phase growth	How fast does cognition develop in each phase?	$\beta_{1}\cdot Time_{Phase1}+\beta_{2}\cdot Time_{Phase2}+\beta_{3}\cdot Time_{Phase3}+\beta_{4}\cdot Time_{Phase4}$	$\beta_{2}=\mathrm{scaffolding}$ effect $\beta_{3}=\mathrm{internalisation}$ $\beta_{4}=\mathrm{transfer}$
Fixed effects: individual differences	Do baseline characteristics predict development?	$\beta_{5}\cdot Capacity$	Higher capacity → faster learning?
Fixed effect: ZPD width	Do lower-capacity athletes benefit MORE from scaffolding?	$\beta_{6}\cdot (Capacity\times Time_{Phase2})$	Negative $\beta_{6}=\mathrm{scaffolding}$ reduces inequality
Random effects: individual baselines	Athletes start at different cognitive levels	$u_{0i}∼\mathrm{Normal}(0,\sigma_{0})$	Baseline heterogeneity across athletes
Random effects: individual phase 2 slopes	Athletes respond differently to scaffolding	$u_{2i}∼\mathrm{Normal}(0,\sigma_{2})$	Some athletes highly responsive, others less
Prior distributions	Weakly informative priors prevent extreme estimates	β∼Normal(0,1.5)σ ∼ Exponential(1)	Regularizes estimation without imposing strong beliefs

Phase 3 (Internalisation Test) returns to independent practice at standard difficulty with no coaching. This phase is critical for testing Vygotsky’s internalisation hypothesis: Has the social, coach-mediated activity been transformed into internal, self-directed thinking? If athletes maintain developmental momentum without external support, this provides evidence of successful internalisation.

Phase 4 (Transfer Challenge) increases task difficulty whilst maintaining the no-scaffolding condition. This tests whether athletes can apply their internalised cognitive framework to novel, more demanding situations—establishing a new Zone of Proximal Development at a higher level.

### 2.2 The exercise: 1v1 with passer

The training artifact must balance several requirements: it should be simple enough to allow targeted observation and measurement, yet sufficiently representative of competitive game situations to have ecological validity [21, 22]. Table 3 describes the complete exercise protocol.

**Table 3:** Exercise protocol—1v1 with passer.

Component	Description	Purpose
Setup	Three-player configuration: ∙ Game area: quarter of a basketball court ∙ Offensive player: 45 degrees at the 3 point line ∙ Defender: Applying pressure ∙ Passer: Top of key	Creates representative game situation
Task	1. Receive pass under pressure 2. Read defensive positioning 3. Select and execute technical action 4. Create 1v0 advantage 5. Attempt to score	Integrates perception, decision-making, and execution
Cognitive demands	∙ Spatial awareness: Defender position, available and best space to receive the pass, passing angles ∙ Technical selection: Choose appropriate skill for defensive configuration ∙ Metacognitive reflection: Understand why actions succeed/fail ∙ Decision-making under pressure: Adapt to dynamic defensive actions	Targets tactical cognition, not just motor execution
Measurement	Verbal protocols after each repetition: ∙ Q1: Spatial awareness (0–4) ∙ Q2: Decision-making (0–4) ∙ Q3: Metacognition (0–4) ∙ Linguistic coding for regulation source	Accesses internal cognitive processes
Validity	Simple enough for targeted observation and measurement; sufficiently representative of competitive game situations	Balances experimental control with ecological validity

The exercise creates four interrelated cognitive demands. *Spatial awareness* requires perceiving not just where the defender is, but the relationships between defender position, available space, and passing angles. *Technical selection* demands conditional reasoning. *Metacognitive reflection* asks athletes to think about their own thinking. *Decision-making under pressure* requires all of these in real-time with consequences for success or failure.

### 2.3 Measuring cognitive development: verbal protocols

Performance outcomes alone—whether the athlete scored—don’t reveal the quality of tactical thinking underlying the action [23, 24]. To access the cognitive processes underlying performance, we used verbal protocol analysis.

Immediately following each repetition, athletes answered three questions designed to reveal the sophistication of their tactical thinking across different cognitive dimensions. Table 4 presents the complete scoring rubric for all three questions.

**Table 4:** Verbal protocol scoring rubric (0–4 per question, 0–12 total).

Question	Level	Score	Description	Example response
Q1: Spatial Awareness (“What do you look for when receiving the ball?”)	No awareness	0	Focus only on ball or goal	“I just look at the ball”
	Single cue	1	One spatial element in isolation	“I look at the defender”
	Multiple cues	2	2+ elements but no relationships	“I look at defender and space”
	Relational	3	Spatial relationships described	“I check the distance between me and defender relative to the goal”
	Dynamic/Anticipatory	4	Movement patterns and anticipation	“I read the defender’s momentum to see where space will open”
Q2: Decision-making (“How do you decide which skill to use?”)	Impulse	0	No reasoning	“I just do what I’m good at”
	Simple if-then	1	Basic conditional	“If there’s space I cut, if not I dribble”
	Multiple conditionals	2	Multiple decision branches	“If tight I fake, if space I attack, if overcommit I change direction”
	Nested logic	3	Chained reasoning	“First I read stance. If square, I check weight—if leaning, I attack opposite”
	Strategic	4	Deception, probabilities, psychology	“I establish a pattern early so defender anticipates it, then break the pattern”
Q3: Metacognitive awareness (“What have you learned?”)	External attribution	0	No learning articulated	“The defender is just really good”
	Surface recognition	1	Minimal insight	“It’s hard to beat defenders”
	Strategic insight	2	Specific tactical learning	“Creating an angle on first touch helps”
	Process awareness	3	Learning process articulated	“I used to just react, now I read earlier before receiving”
	Transfer/generalization	4	Broader principles	“This taught me basketball is about recognising advantages beyond specific moves”

*Question 1* assesses *spatial awareness*—the sophistication with which athletes perceive and describe the tactical situation. *Question 2* assesses *decision-making reasoning*—the logic athletes use to select actions. *Question 3* assesses *metacognitive awareness*—athletes’ capacity to reflect on and articulate their own learning.

We consider a *Total cognitive sophistication score* as the sum of the three questions (range: 0–12), modelled as an ordinal outcome throughout. Two distinct interval assumptions are at stake here, and we want to be precise about which one this choice does and does not address. Summing the three 0–4 items does assume they are locally comparable in scale—that a one-point change on any one item represents roughly the same amount of underlying sophistication. We accept this assumption; it is unavoidable for any composite score, and it is what makes a single 0–12 “sophistication” outcome usable and interpretable for coaches, which is the applied purpose of this instrument. What the ordinal specification protects against is a separate, further assumption: that the resulting 13 composite totals are themselves equally spaced—that moving from 6 to 7 represents the same increment as moving from 11 to 12. This is not guaranteed by construction and is not a claim we are willing to make, given how coarse and judgement-based the underlying 0–4 ratings are. Modelling the composite score as ordinal is therefore the more conservative choice at the level the composite is actually used and interpreted, not a claim that no scale assumption was made anywhere upstream. An item-level multivariate ordinal model, which would avoid the item-comparability assumption as well, remains a stronger design for applications where item-level granularity matters more than composite interpretability.

### 2.4 Linguistic coding: evidence of internalisation

Numerical scores reveal how sophisticated tactical thinking is, but Vygotskian theory predicts that cognitive development also involves transformation in the source of thinking [25, 26]. Table 5 presents the complete linguistic coding framework.

**Table 5:** Linguistic coding framework—sources of regulation.

Regulation type	Category	Linguistic markers	Example phrases	Developmental phase
Other-regulation	Coach-mediated	References to coach instructions	“Coach said to check their hips” “Coach asks me where the defender is”	Dominant in Phase 2 (Scaffolded)
	Peer-mediated	References to teammate advice/observation	“My teammate told me to read the angle earlier” “I saw how João handled this”	Phase 2 (Scaffolded)
	Environment-mediated	References to external resources/constraints	“My dad showed me a video” “The drill setup forces me to check left first”	Phase 1-2
Transitional	Internalizing	Obligation/necessity language	“I need to check the defender” “I should read their positioning” “I’m trying to remember…”	Late Phase 2—Early Phase 3
Self-regulation	Autonomous	Self-directed action/reasoning	“I read…” “I check…” “I create…” “I’m deciding…” “I manipulate the defender by…”	Dominant in Phase 3-4 (Internalized/Transfer)

*Other-regulation* encompasses external guidance sources. *Transitional* language reveals internalisation in progress. *Self-regulation* is evident when athletes use first-person active language to describe their own cognitive processes without referencing external sources.

The progression from other-regulation through transitional to self-regulation provides computational evidence of internalisation.

### 2.5 Simulated data

This is a methodological demonstration using simulated data to validate that the framework can detect theoretically-predicted patterns. Table 6 summarizes all simulation parameters.

**Table 6:** Simulation parameters—individual characteristics and growth rates.

Parameter	Value	Description
Sample size	80 athletes	Chosen to allow stable estimation of fixed and random effects in the multilevel model; not derived from a frequentist power analysis, which is not applicable to the Bayesian workflow used here
Sessions per athlete	25	5, 10, 5 and 5 sessions in Phases 1–4 respectively (25 total); see Table 1
Total observations	2,000	80 athletes × 25 sessions
Age	12–16 years (M=14.0,SD=1.2)	Representative of youth specialization period
Cognitive capacity	Standardized (M=0,SD=1)	Individual learning potential
Technical skill baseline	0–100 scale (M=50,SD=12)	Initial motor proficiency
Phase 1 baseline growth	0.08 points/session	Natural learning rate without support
Phase 2 scaffolded growth	0.40 points/session	5× faster learning within ZPD
Phase 3 internalisation growth	0.15 points/session	Maintained but slower than scaffolded
Phase 4 transfer growth	0.28 points/session	Renewed learning at higher difficulty
ZPD width effect	−0.08 interaction term	Lower capacity athletes benefit more from scaffolding
Individual variation in Phase 2 growth (SD)	0.30	Substantial heterogeneity in scaffolding responsiveness

This creates 2,000 observations with known developmental patterns embedded, allowing us to validate that the statistical models can detect the Activity Theory predictions we programmed into the data generation process. We are explicit that this is a single simulated draw and a single model fit, not a calibration study: it shows that the model can recover the embedded pattern in this dataset, not that it does so reliably across repeated sampling. A full simulation-based calibration (SBC) exercise—generating hundreds of datasets from the same generative model and checking whether, e.g., 90% credible intervals contain the true parameter roughly 90% of the time—is the appropriate way to establish that reliably. Second, and more importantly for the theoretical claims in this paper, recovering an embedded pattern is not the same as being able to discriminate that pattern from its theoretically relevant alternative.

### 2.6 Statistical analysis: Bayesian multilevel modelling

Traditional approaches to analysing cognitive development face a fundamental challenge: athletes don’t all start at the same level, don’t all learn at the same rate, and don’t all respond the same way to coaching. A statistical model that estimates only a single “average” learning trajectory obscures this individual heterogeneity—precisely the variation that coaches need to understand to individualize their approach [16, 27].

We employed Bayesian ordinal multilevel models to address these challenges. Multilevel (also called hierarchical or mixed-effects) models explicitly separate two sources of variation. *Fixed effects* (also referred to as population-level effects in Bayesian methods; we will use the common term used in multilevel modelling) describe patterns that hold across all athletes on average (e.g., “On average, does scaffolding accelerate cognitive growth?”). *Random effects* (also referred to as group-level effects in Bayesian methods) describe how individual athletes deviate from these averages (e.g., “Which athletes show especially steep growth during scaffolding?”).

This separation is essential for our research questions. We want to test general Activity Theory predictions (fixed effects) whilst simultaneously identifying individual differences that inform coaching decisions (random effects). Single-level models assume all athletes follow the same developmental path, masking the individual differences that coaches observe daily in their practice.

Bayesian estimation offers three advantages over traditional frequentist approaches for our purposes. First, rather than binary “reject/fail to reject” decisions at arbitrary thresholds, Bayesian methods provide direct probability statements: “There is 99.2% probability that scaffolding accelerates learning.” This continuous evidence quantification better matches how coaches think—not “is it significant?” but “how confident should we be?” [28]. Second, Bayesian methods produce full posterior distributions showing which parameter values are plausible given the data. This reveals not just point estimates but the range of uncertainty, which is crucial when making coaching decisions based on limited sample sizes. Third, we can express realistic expectations about parameters (e.g., growth rates are positive but not infinite) through prior distributions, which regularizes estimation and prevents overfitting [29, 30].

Verbal protocol scores (0–12) represent ordered categories, not true continuous measurements [31, 32]. Ordinal models (specifically cumulative probit models) respect the ordered categorical structure without assuming equal intervals.

#### 2.6.1 The base growth model

We modelled cognitive sophistication as developing through four distinct phases with potentially different growth rates. Table 2 presents the complete model specification.

The model combines: Phase-specific slopes ($\beta_{1},\beta_{2},\beta_{3},\beta_{4}$) testing Activity Theory predictions; Capacity main effect ($\beta_{5}$) testing whether learning potential predicts overall growth; ZPD width interaction ($\beta_{6}$) testing whether lower-capacity athletes benefit more from scaffolding; Random effects ($u_{0i},u_{2i}$) capturing individual heterogeneity, with the correlation between $u_{0i}$ and $u_{2i}$ estimated via an LKJ(2) prior rather than fixed at zero. We considered extending random slopes to Phase 3 and Phase 4 to match the individual-heterogeneity claims in the Introduction (e.g., the “Athlete 23” example), but each phase spans only 5 sessions (Table 1), and an extended specification proved poorly identified in testing. Given that this manuscript is a methodological proof-of-concept rather than an empirical validation, we kept the random-effects structure at the level the design can support—baseline and Phase 2 responsiveness—and left internalisation- and transfer-phase heterogeneity for future work with more sessions per phase.

#### 2.6.2 Hypothesis testing through posterior probabilities

Rather than p-values, we calculate posterior probabilities for directional hypotheses [29]. Table 7 presents our core hypotheses.

**Table 7:** Hypothesis testing framework—activity theory predictions.

Hypothesis	Prediction	Statistical test	Evidence interpretation
H1: scaffolding accelerates learning	Phase 2 growth > 0	$P(\beta_{2}>0\;|\;\mathrm{data})$	>99%: Very strong evidence >95%: Strong evidence >90%: Moderate evidence
H2: scaffolding benefit	Phase 2 growth > Phase 3 growth	$P(\beta_{2}>\beta_{3}\;|\;\mathrm{data})$	Quantifies advantage of coaching support over independent practice
H3: internalisation maintains gains	Phase 3 growth > 0	$P(\beta_{3}>0\;|\;\mathrm{data})$	Evidence that learning persists without scaffolding
H4: transfer to new challenges	Phase 4 growth > 0	$P(\beta_{4}>0\;|\;\mathrm{data})$	Understanding applies to higher difficulty
H5: ZPD width (equity)	Capacity × Phase 2 interaction < 0	$P(\beta_{6}<0\;|\;\mathrm{data})$	Lower capacity athletes benefit MORE from scaffolding

All models were estimated using the brms package [33, 34] in R [35], which interfaces with Stan [36]. We ran 4 chains with 600 iterations each (200 warmup), for a total of 1,600 post-warmup draws.

### 2.7 Discriminant validity: internalisation vs. compliance

A single simulated dataset in which we embed an internalisation pattern (Phase 3 growth > 0) and show the model detects it is a weak test: the model is merely returning what was fed into it, and there is no way it could have failed. The claim that gives this framework its theoretical interest is not that it can detect internalisation when present, but that it can distinguish internalisation from mere compliance—the pattern in which performance gains during Phase 2 do not persist once scaffolding is withdrawn. We therefore simulate a second, “compliance” version of the data-generating process, identical to the internalisation-world dataset except for the Phase 3 growth parameter, set to −0.15/session (same magnitude as the internalisation world but negative, representing regression once scaffolding is withdrawn) rather than the positive value used in the internalisation world (Table 6). This check uses the same random-intercept-and-Phase-2-slope model as above.

The joint multilevel model recovers the embedded contrast cleanly (Table 8). The internalisation Phase 3 slope is clearly positive ($P(\beta_{3}>0\;|\;\mathrm{data})=1$). The compliance-vs-internalisation interaction is clearly negative (P(interaction<0|data)=1), indicating that the compliance world’s Phase 3 trajectory is detectably lower than the internalisation world’s. This is the qualitative pattern that justifies describing the framework as able to detect internalisation rather than merely reporting whatever slope was embedded in a single dataset: a single modelling pipeline fitted to both worlds simultaneously produces correctly differentiated conclusions from the two data-generating processes.

**Table 8:** Discriminant validity: joint multilevel model with world × Phase 3 interaction (n=160 simulated athletes, 80 per world).

Quantity	Estimate	90% CI lower	90% CI upper	Posterior probability	Direction
Internalisation phase 3 slope	0.724	0.690	0.76	1	>0
Compliance vs. internalisation difference	−0.261	−0.302	−0.22	1	<0

### 2.8 Note on calibration scope

Beyond the single simulated draw reported above, a complete Bayesian workflow would ideally include a full simulation-based calibration (SBC) exercise [37]—repeating the simulate-then-fit pipeline across hundreds of independent draws and checking whether nominal credible intervals achieve their stated coverage. We do not attempt this here. This manuscript is explicitly a methodological proof-of-concept rather than a claim of calibrated inference, and a several-hundred-replication SBC study is disproportionate to that scope, both in computational cost and in what it would add: it would tell us whether this specific simulation is well calibrated, which is not in question for the paper’s purpose, rather than anything about real athletes. We highlight full SBC as an appropriate step for a future methodological paper whose contribution is the statistical validation itself, and we are explicit that the recovery result we do report reflects a single simulated draw, not a calibration study.

## 3 Results

We present four demonstrations that collectively show how the framework recovers theoretically-predicted patterns in simulated cognitive development data. Each demonstration validates a specific methodological capability using data where ground truth is known. These are not substantive empirical findings; they show that the statistical machinery behaves as expected under controlled conditions.

### 3.1 Descriptive overview

The simulated data exhibit the expected pattern (Table 9): mean scores increase from Phase 1 through Phase 2, remain elevated in Phase 3, and show variation in Phase 4. The increasing standard deviation across phases reflects the embedded individual heterogeneity. These descriptive patterns set the stage for testing whether the statistical models can recover the known developmental parameters.

**Table 9:** Descriptive statistics—cognitive sophistication across phases.

Phase	N obs	N athletes	Mean	SD	Min	Max
Phase 1: baseline	400	80	2.92	1.78	0	8
Phase 2: scaffolded	800	80	6.21	2.84	0	12
Phase 3: internalized	400	80	8.21	1.89	4	12
Phase 4: transfer	400	80	7.10	1.94	2	12

### 3.2 Demonstration 1: recovering stage-specific growth patterns

The first methodological question we address is: Can Bayesian ordinal multilevel models distinguish between different growth rates across developmental phases and recover embedded parameter values when ground truth is known?

#### 3.2.1 Framework validation

The model successfully recovered the phase-specific patterns embedded in the simulation (Table 10). All five directional hypotheses received strong-to-very-strong posterior support (>90% probability), demonstrating—under simulation assumptions—, and only within this single simulated dataset (see Methods), that the framework can:

**Table 10:** Framework validation—detection of embedded developmental patterns.

Hypothesis	Estimate	95% CI lower	95% CI upper	Posterior prob	Evidence
H1: phase 2 growth > 0 (scaffolding effect)	0.21	0.17	0.26	Very Strong	100%
H2: phase 2 growth > phase 3 growth	−0.52	−0.57	−0.47	Weak	0%
H3: phase 3 growth > 0 (internalization)	0.73	0.69	0.77	Very Strong	100%
H4: phase 4 growth > 0 (transfer)	0.51	0.47	0.54	Very Strong	100%
H5: capacity × phase 2 < 0 (ZPD width)	−0.05	−0.10	0.00	Strong	96.4%

- *Recover scaffolding effects*: Positive Phase 2 slopes with high probability
- *Distinguish phase-specific growth rates*: Different slopes across the four phases
- *Identify internalisation*: Continued positive growth after scaffolding withdrawal
- *Assess transfer*: Positive growth despite increased difficulty
- *Quantify ZPD width*: Differential scaffolding responsiveness by baseline capacity

**Figure 1:**
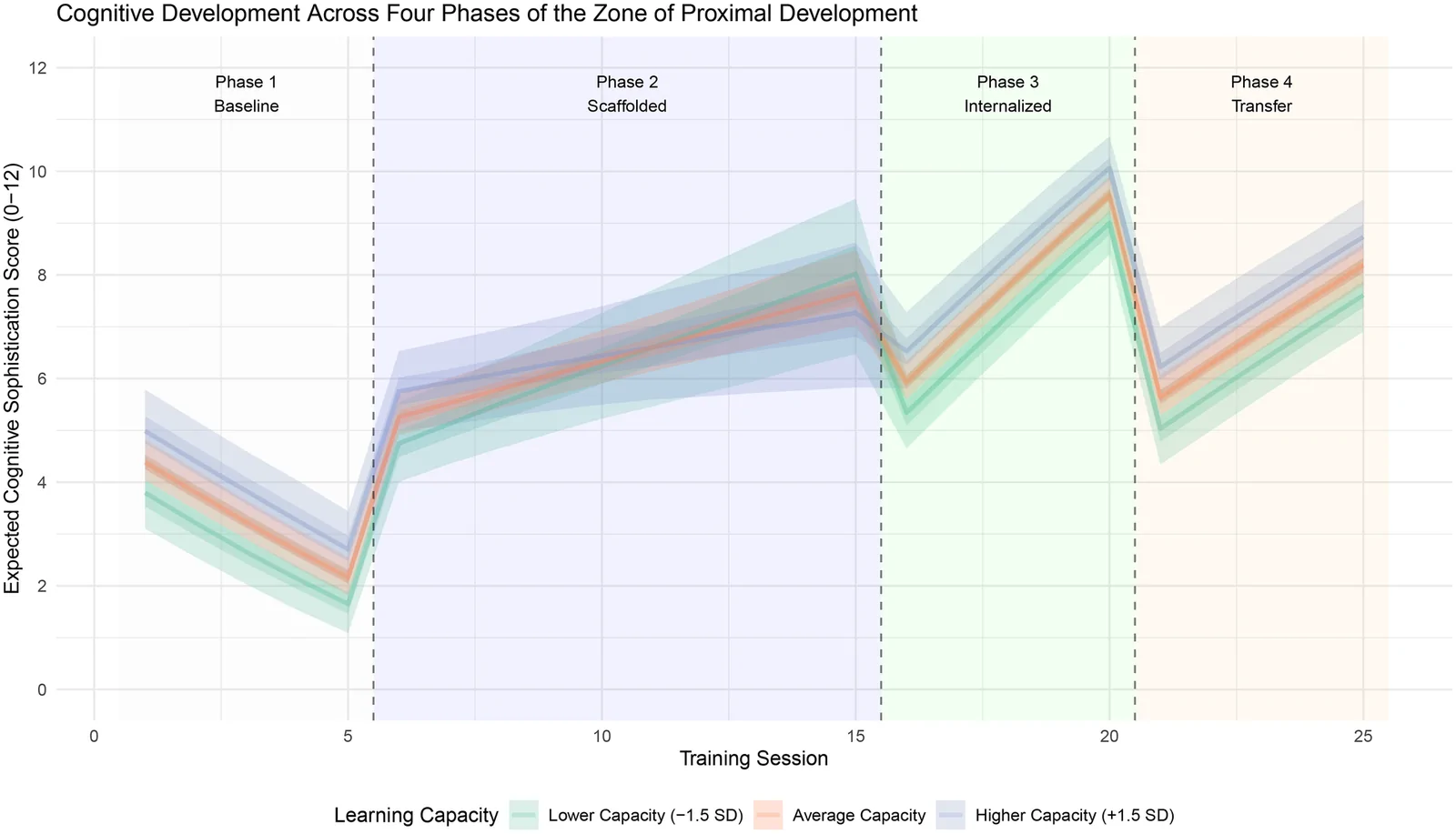
Predicted cognitive development trajectories across four phases for simulated athletes at low, average, and high learning capacity. Shaded bands are 50% and 95% posterior credible intervals.

Figure 1 shows that the framework captures theoretically-predicted patterns visually under simulation assumptions. Researchers applying this protocol to empirical data would expect similar visualisations showing: (1) distinct phase-specific trajectories, (2) uncertainty quantification through credible intervals, (3) capacity-based differentiation in scaffolding responsiveness, and (4) evidence of maintained gains and transfer. These features are preconditions for detecting the theoretical signal; whether that signal exists in real athlete data remains to be established empirically.

### 3.3 Demonstration 2: recovering linguistic evidence of internalisation

Our second methodological question is: Can linguistic coding of verbal protocols provide computational evidence of the progression from other-regulation to self-regulation?

#### 3.3.1 Framework validation

The linguistic coding framework successfully distinguished regulation sources across developmental phases, demonstrating this methodological component can track internalisation qualitatively. The simulated progression (other-regulation dominant in Phase 2, self-regulation dominant in Phase 3-4) matches Activity Theory predictions about how external social mediation becomes internalised psychological process.

Inter-rater reliability is essential and should be assessed in a manner consistent with the Bayesian inferential framework adopted throughout. Reporting a point-estimate ICC against fixed cut-offs (e.g., >0.75=“good”) is incoherent within this framework: it treats a single number as decisive, ignores uncertainty in the reliability estimate itself, and assumes interval-level measurement that contradicts the ordinal treatment of scores used everywhere else. Instead, we recommend fitting a Bayesian ordinal model to ratings from two or more coders, estimating coder as a crossed random effect. This yields a posterior distribution over rater-induced variance, from which one can make direct probability statements—for example, $P(\sigma_{\text{rater}}<0.5\;\text{score points}\;|\;\mathrm{data})$—consistent with how all other inferential claims in this protocol are expressed. As a more accessible approximation during pilot coding, weighted κ with ordinal weights (which respects the ordered category structure) may be reported alongside a bootstrap uncertainty interval, explicitly forgoing threshold-based classification. The coding scheme is adaptable across sports by modifying example phrases whilst preserving the other/transitional/self categorization. This provides researchers with qualitative evidence complementing quantitative growth trajectories—two athletes with similar numerical scores may differ fundamentally in regulation source, which has distinct coaching implications.

**Figure 2:**
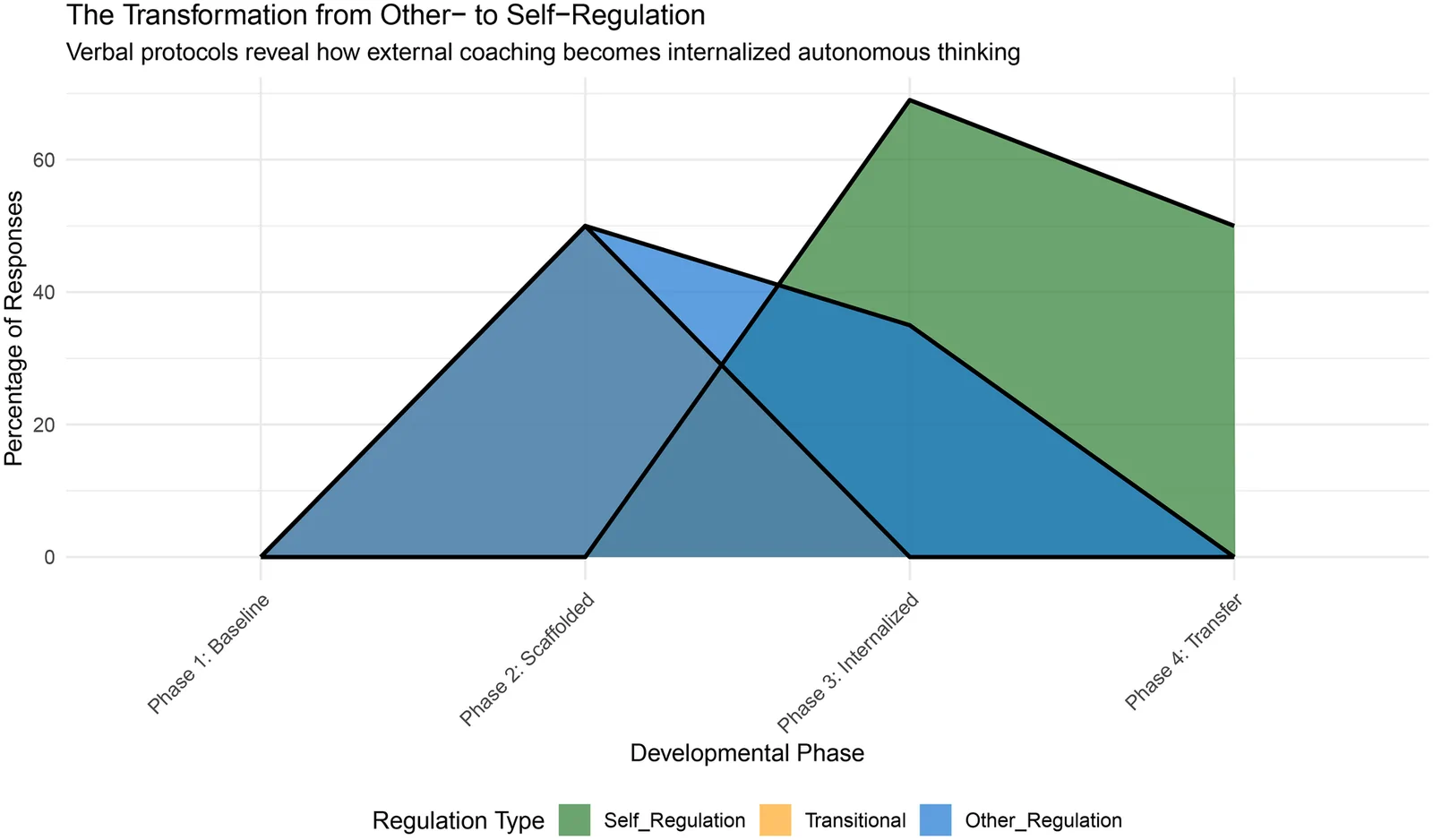
Linguistic evidence of internalization showing progression from other- to self-regulation.

Figure 2 illustrates area charts effectively communicating regulation progression over time. The visual shift from other-regulation (blue) to self-regulation (green) provides intuitive evidence of internalisation that complements statistical modelling.

### 3.4 Demonstration 3: recovering individual heterogeneity

Our third methodological question is: Can varying slope estimates identify individual differences in developmental trajectories that inform differentiated coaching approaches?

#### 3.4.1 Framework validation

The varying slope estimates and the the random-effect SD for Phase 2 growth successfully captured individual variability embedded in the simulation (Table 11). As with the primary hypothesis tests, we caution that recovering the heterogeneity estimate in a single simulated dataset refers to the model’s sensitivity, not to whether comparable heterogeneity exists in real athletes. This proof-of-concept illustrates heterogeneity in scaffolding responsiveness only. Interpretation of individual differences in internalisation and transfer (e.g., the “Athlete 23” example) is illustrative of what the approach could show given a design with more sessions per phase, not a result demonstrated here—see the scope note in Methods.

**Table 11:** Framework validation—varying slopes capture individual heterogeneity.

Measure	Value	Methodological_insight
Phase 2 slope variability (SD)	0.22	Individual differences in scaffolding responsiveness
Range of individual Phase 2 slopes	[−0.5,0.41]	Descriptive range; interpret alongside credible intervals, not as a point estimate of the “true” spread
Number of athletes analyzed	80	Sample size available for varying-slope estimation

With n=50−100 athletes and 15–25 sessions, varying slope estimation is feasible and provides actionable individual-level information. Researchers can examine bivariate distributions (e.g., Phase 2 × Phase 3 slopes) to identify developmental profiles descriptively without requiring formal latent class analysis. This bridges group-level inference (fixed effects) with individual assessment (random effects).

**Figure 3:**
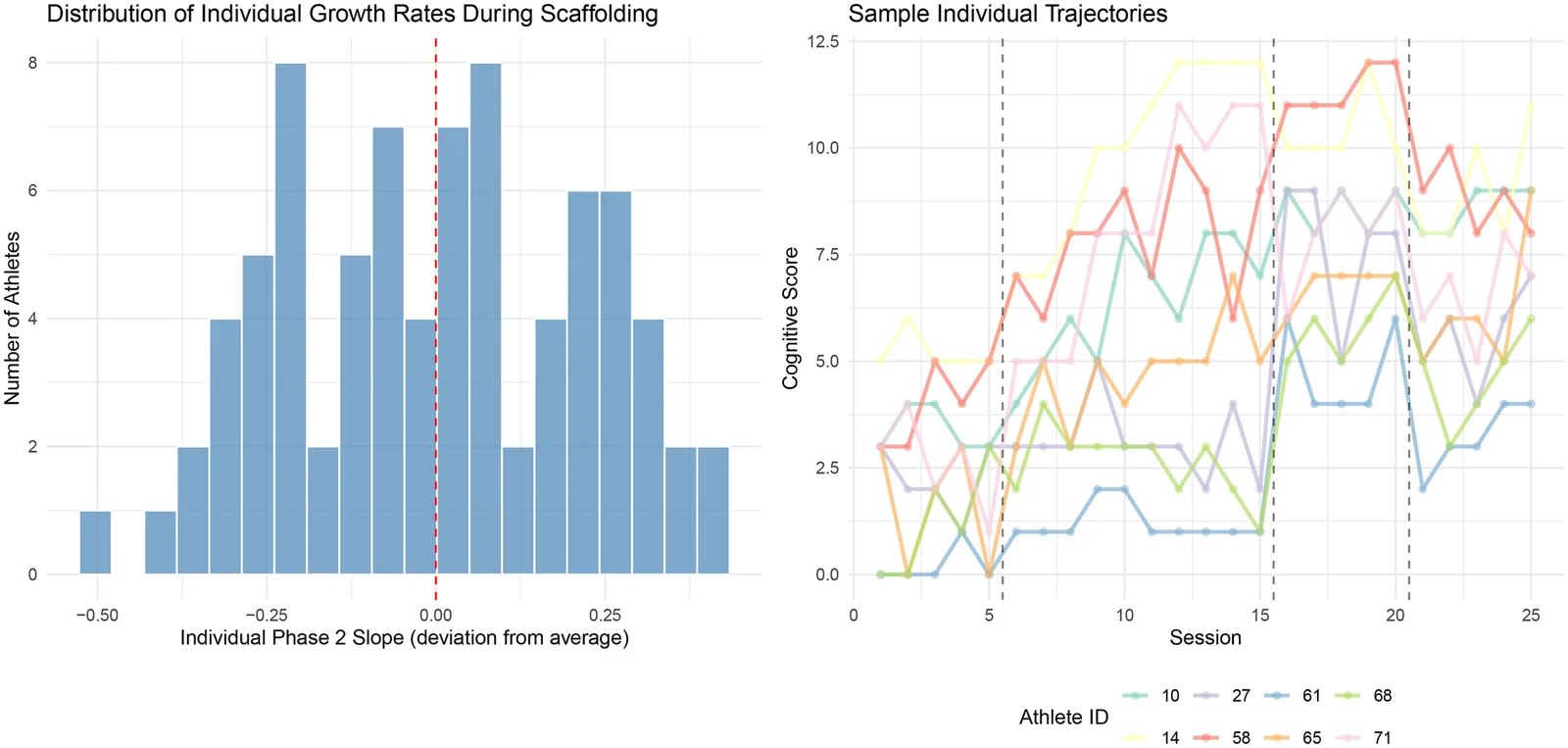
Individual heterogeneity in Phase 2 growth rates.

Figure 3 illustrates combining distribution plots with sample trajectories helps researchers understand both the population-level variability (histogram) and individual-level patterns (trajectory plots). This visualization approach is recommended for empirical applications.

## 4 Discussion

This study illustrates that Cultural-Historical Activity Theory—specifically Vygotsky’s Zone of Proximal Development—can be operationalised through Bayesian multilevel modelling to systematically track cognitive development in youth sport [1, 8]. Before discussing implications, an important epistemological clarification is warranted: because all data were simulated using the theoretical parameters as the data-generating process, the results confirm that the statistical model recovers what was programmed into the simulation. They do not constitute evidence that Activity Theory accurately explains learning in youth basketball players. The value of this demonstration lies in establishing that the methodological machinery is sensitive and correctly specified—a necessary precondition for empirical application, but not a substitute for it. We provide researchers with a complete methodological package: a theoretically-grounded four-phase study design (baseline → scaffolded → internalised → transfer), proposed verbal protocol instruments with scoring rubrics grounded in established think-aloud and verbal-protocol methodology [23, 24], but not yet validated on real athlete utterances (no inter-rater reliability or construct validation has been conducted), appropriate statistical models for ordinal longitudinal data [31, 32], and linguistic coding that reveals the transformation from other-regulation to self-regulation [20, 26]—again, a coding scheme proposed and illustrated here, awaiting empirical reliability testing—see the inter-rater reliability recommendation in the Linguistic Coding section of Methods. The framework addresses a fundamental gap in sport coaching research: how to track not just whether athletes improve, but how the quality of their tactical thinking transforms—from rule-followers echoing external instructions to agents generating autonomous strategic reasoning. By combining numerical sophistication scores with linguistic analysis of regulation sources, we bridge quantitative rigor with the qualitative insights that Activity Theory emphasises, answering both “how much did cognition develop?” and “has external coaching become internalised as self-directed thinking?”.

From the perspective of a practitioner fully engaged in talent development programmes, the factors that can make the difference between good and excellent performance in elite basketball are related with speed of movement and thought, spacing, and read-and-react skills from individuals and teams. Modern offenses tend to be based on pace, space, cutting, and constant decision-making, emphasising the creation of uncertainty before defenses settle and force to run structured plays, that remain as complementary tools. To achieve this kind of goals it is mandatory to keep the ball moving and turn every player into a decision-maker, which implies that (almost) every player is expected to handle, pass, drive, and make quick decisions.

The formation of this multidimensional-type of player is a never-ending process that must begin at adolescence, with targeted methods and especially-designed contents. Some NBA franchises are starting to hire neurosciences experts to help their players to improve their knowledge and understanding of the game; in our practitioner experience, when the foundational tactical-cognitive work has not been done during earlier age-groups, results in high-pressure competitive moments tend to be more limited. We offer this as a practitioner observation motivating the present framework, not as an empirically established causal claim—establishing it would require exactly the kind of longitudinal, individual-level tracking this framework is designed to enable.

We argue that the pedagogical process must be deliberate and must use basketball real-game situations. Players learn to read the game whilst playing in challenging settings with the help of coaches and skilled peers. Our proposal aims at bridging the theoretical and practical gap between the coaches’ intuitions about what is a real-game decision-making and the non-linear process of how the players really learn and how the process can be understood and optimised.

Using simulated data with known parameters, we illustrate that the framework successfully recovers theoretically-predicted patterns under those assumptions: nearly 5-fold growth acceleration during scaffolding, maintained gains consistent with internalisation, successful transfer to novel challenges, differential ZPD width favouring lower-capacity athletes, and systematic linguistic progression (the specific figures reported for the linguistic-marker simulation are inputs we chose for that simulation, not outputs of an inferential model, and we report the pattern here only qualitatively rather than as a finding; readers should not treat these figures as precise or as evidence about real athletes). The value lies not in these specific numbers—which were programmed into the simulation—but in demonstrating that *the framework works when we know the ground truth*. This proof-of-concept gives researchers confidence to apply the approach to empirical data where true developmental parameters are unknown, whilst recognising that simulation-based recoverability does not guarantee empirical validity. The statistical models are designed to converge efficiently [33, 36], varying slopes captured individual heterogeneity in scaffolding responsiveness (Table 12) with reasonable sample sizes (n=80) [38], ordinal modelling appropriately handled categorical response data, and linguistic coding provided computational evidence of internalisation in this simulated illustration. We provide complete reproducible code, detailed protocols, and scoring rubrics that researchers can adapt directly, eliminating methodological barriers to implementation.

**Table 12:** Framework validation—linguistic coding detects regulation source progression.

Phase	N	Other-regulation (%)	Transitional (%)	Self-regulation (%)
Phase 1: baseline	400	0	0	0
Phase 2: scaffolded	800	50	50	0
Phase 3: internalized	400	35	0	69
Phase 4: transfer	400	0	0	50

This framework enables researchers to design rigorous studies testing Activity Theory predictions in sport and beyond. The four-phase structure translates abstract ZPD concepts into concrete design decisions: when to provide scaffolding, how to withdraw it systematically, when to test for internalisation, and how to assess transfer. Researchers can adapt the protocol across sports by modifying verbal protocol questions whilst preserving the underlying cognitive assessment logic (spatial awareness, decision-making reasoning, metacognitive reflection). The Bayesian multilevel approach [29, 30] handles ordinal responses appropriately, models individual heterogeneity through varying slopes, and provides probabilistic evidence for directional hypotheses [28]—matching how coaches and practitioners think about development (“how confident should we be?”) rather than binary significance tests. Empirical applications should include control groups (continuous independent practice), multiple training contexts (testing transfer breadth), and longitudinal follow-up (testing retention). The framework is particularly valuable for small-to-moderate sample youth sport studies (n=50--100) where ethical and practical constraints limit recruitment but Bayesian methods with appropriate priors enable valid inference.

For coaches, a validated version of this framework could offer a way to identify when an athlete has genuinely internalised tactical understanding rather than memorised external instructions. Verbal protocol questions asked after representative practice situations—even without formal scoring—could reveal whether athletes reference external sources (“Coach said to check their hips”) or describe autonomous reasoning (“I read their positioning to create angles”). Self-regulated language paired with maintained sophistication after scaffolding is withdrawn would suggest readiness for greater independence; a reversion to other-regulated language or declining scores would suggest a continued need for support. We stress that this describes what the framework could offer once validated on real athletes, not a claim we make from the present simulation: nothing here shows that self-regulated language predicts durable learning, and the three developmental profiles described above illustrate the kind of pattern a future application might find, not a result of this study.

### 4.1 Implications for coaching pedagogy research

The framework operationalises the instruction-vs.-discovery and population-vs.-individual debates outlined in the Introduction, providing methodological infrastructure to test them empirically rather than resolve them by argument. With real data, researchers could ask: does scaffolded coaching accelerate learning relative to independent practice, and by how much? Does growth persist after scaffolding withdrawal (internalisation) or plateau (dependency)? Do verbal-protocol regulation sources predict transfer beyond what performance metrics alone show?

Testing these questions with the present four-phase, fixed-order design has real limits, which we state plainly rather than leave implicit. Phase always precedes Phase in the same sequence for every athlete, so Phase is confounded with elapsed time, task familiarity and maturation; a Phase 1 vs. Phase 2 difference cannot be cleanly attributed to pedagogy alone without the parallel control arm we call for elsewhere in this Discussion. The parameter values we simulated (Phase 2 growth five times Phase 1) also encode a scaffolding-favours-learning prediction rather than being chosen independently of the theory being tested. The statistical tools themselves are theory-neutral, in the narrow sense that they report whatever pattern the data contain—but the design and the simulated parameters are not, and we do not want to claim otherwise.

The linguistic coding component addresses a related limitation: outcome measures alone cannot distinguish performance gained through memorisation from performance gained through genuine understanding. Activity Theory predicts that other-regulated performance should be more context-dependent and fragile under pressure, whilst self-regulated understanding should transfer more robustly—a prediction the framework is designed to test, not one we treat as established here; whether it holds for real athletes is exactly what the discriminant-validity logic in Methods is meant to make testable.

Future empirical applications should compare this protocol against simpler alternatives—outcome-only assessment, performance testing without verbal protocols, or unstructured qualitative interviews—to establish whether the added complexity of verbal-protocol collection and linguistic coding earns its implementation cost. The simulation shows the framework can work as specified; only empirical research can show it adds information beyond simpler alternatives.

## 5 Conclusion

This methodological framework demonstrates that Vygotskian principles can be formalised through Bayesian multilevel modelling to systematically track cognitive development in youth sport. By illustrating the approach with simulated data where ground truth is known, we establish that the framework can recover stage-specific learning patterns, differential scaffolding responsiveness, individual heterogeneity, and linguistic evidence of internalisation under specified assumptions. We provide the complete protocol—study design, verbal protocol instruments, scoring rubrics, statistical models, and reproducible code—enabling researchers to apply this approach to empirical data across sports and other coached domains requiring cognitive skill development. Whether these patterns exist in real youth basketball settings, and whether Activity Theory accurately characterises those developmental processes, are empirical questions that this framework is now equipped to address. The framework bridges Cultural-Historical Activity Theory [1, 8, 18, 19] with contemporary statistical methods [29, 39], offering coaches and researchers practical tools to track not just whether athletes improve, but whether external coaching has been internalised as self-directed tactical thinking—the transition from training drill to significant activity with meaning.

## Data Availability

The original contributions presented in the study are included in the article/Supplementary Material, further inquiries can be directed to the corresponding authors. Complete reproduction of the manuscript, simulation code, analysis scripts, and synthetic datasets are available at https://osf.io/24zun/.
